# Structure and Function of HLA-A*02-Restricted Hantaan Virus Cytotoxic T-Cell Epitope That Mediates Effective Protective Responses in HLA-A2.1/K^b^ Transgenic Mice

**DOI:** 10.3389/fimmu.2016.00298

**Published:** 2016-08-08

**Authors:** Ying Ma, Linfeng Cheng, Bin Yuan, Yusi Zhang, Chunmei Zhang, Yun Zhang, Kang Tang, Ran Zhuang, Lihua Chen, Kun Yang, Fanglin Zhang, Boquan Jin

**Affiliations:** ^1^Department of Immunology, The Fourth Military Medical University, Xi’an, China; ^2^Department of Microbiology, The Fourth Military Medical University, Xi’an, China; ^3^Institute of Orthopaedics of Xijing Hospital, The Fourth Military Medical University, Xi’an, China

**Keywords:** Hantaan virus, HLA-A*02, cytotoxic T-cell epitope, crystal structure, CD8^+^ T-cell response, HLA-A2.1/K^b^ transgenic mice

## Abstract

Hantavirus infections cause severe emerging diseases in humans and are associated with high mortality rates; therefore, they have become a global public health concern. Our previous study showed that the CD8^+^ T-cell epitope aa129–aa137 (FVVPILLKA, FA9) of the Hantaan virus (HTNV) nucleoprotein (NP), restricted by human leukocyte antigen (HLA)-A*02, induced specific CD8^+^ T-cell responses that controlled HTNV infection in humans. However, the *in vivo* immunogenicity of peptide FA9 and the effect of FA9-specific CD8^+^ T-cell immunity remain unclear. Here, based on a detailed structural analysis of the peptide FA9/HLA-A*0201 complex and functional investigations using HLA-A2.1/K^b^ transgenic (Tg) mice, we found that the overall structure of the peptide FA9/HLA-A*0201 complex displayed a typical MHC class I fold with Val2 and Ala9 as primary anchor residues and Val3 and Leu7 as secondary anchor residues that allow peptide FA9 to bind tightly with an HLA-A*0201 molecule. Residues in the middle portion of peptide FA9 extruding out of the binding groove may be the sites that allow for recognition by T-cell receptors. Immunization with peptide FA9 in HLA-A2.1/K^b^ Tg mice induced FA9-specific cytotoxic T-cell responses characterized by the induction of high expression levels of interferon-γ, tumor necrosis factor-α, granzyme B, and CD107a. In an HTNV challenge trial, significant reductions in the levels of both the antigens and the HTNV RNA loads were observed in the liver, spleen, and kidneys of Tg mice pre-vaccinated with peptide FA9. Thus, our findings highlight the ability of HTNV epitope-specific CD8^+^ T-cell immunity to control HTNV and support the possibility that the HTNV-NP FA9 peptide, naturally processed *in vivo* in an HLA-A*02-restriction manner, may be a good candidate for the development HTNV peptide vaccines.

## Introduction

As widespread emerging zoonotic pathogens, hantaviruses, which belong to the *Bunyaviridae* family, have significant and growing impacts on global public health (1). Murine-borne hantavirus transmissions cause more than 200,000 cases annually of human disease worldwide and a significant portion of the world’s population is at risk of being infected (2). Some hantaviruses, such as the Hantaan virus (HTNV) in Asia, the Dobrava virus and Puumala virus (PUUV) in Europe, and the Seoul virus worldwide, can lead to hemorrhagic fever with renal syndrome (HFRS) in humans, and other hantaviruses, such as the Sin Nombre virus (SNV) and the Andes virus (ANDV) in North and South America directly cause human hantavirus pulmonary syndrome (HPS) (3–5). Infection by any of the serotypes can lead to a broad spectrum of outcomes, ranging from asymptomatic infection to acute fever and hemorrhage, and even life-threatening shock and acute kidney or lung injury (6). In China, a total of 1,625,002 HFRS cases and 46,968 deaths were reported during the years of 1950–2014 with a case-fatality rate as high as 15% (6). Over 90% of the total global HFRS cases that arose in China were caused by infection with the HTNV strain, the prototype member of the hantavirus family (7–9). In the U.S., based on the Centers for Disease Control and Prevention report, the total HPS cases from the years of 1993–2015 reached 659, with a higher case-fatality rate of ~36%, thereby raising the concern of the World Health Organization. This situation is further exacerbated by the periodic emergence of novel species of hantaviruses, leading to a disruption of the healthcare systems in epidemic areas and a major health concern worldwide (10–12). Although some live attenuated or inactive vaccines have been used in some parts of the world and have partially prevented hantaviruses infections, there remains an urgent need for more effective therapeutic or prophylactic vaccines to better control the epidemic situation (13, 14).

The negative-sense RNA genome encoding two structure proteins, nucleocapsid protein (NP) and envelope glycoprotein, give rise to the primary antigenicity of HTNV. The HTNV-NP has been demonstrated as highly immunogenic and conservative, responsible for vigorous cellular and humoral immune responses (15), and the heterodimers of mature HTNV glycoproteins, Gn and Gc, are considered the main sources of neutralizing antibody production in humans (16). It has been suggested that the immunological mechanisms for viral clearance are related closely with T-cell-mediated immune responses. This notion is supported by evidence that hantavirus-specific T cells play a critical role in the control of virus infection (17–19). Our previous results have demonstrated that the T-cell response during the acute stage of HFRS in patients is characterized by vigorous cytotoxic T cell (CTL) responses and multifunctional T helper (Th) cell responses against HTNV infection (20, 21). Patients with milder severities of HFRS typically mount vigorous HTNV-specific CD4^+^ and CD8^+^ T-cell responses directed against the epitopes of HTNV, whereas patients with severe or critical HFRS tend to have weak and narrowly focused T-cell responses (20–22). Moreover, we also reported that CD8^+^ T cells specific to the immunodominant epitope of HTNV-NP, the aa129–aa137 epitope (FVVPILLKA, FA9), was restricted by human leukocyte antigen (HLA)-A*02 and displayed polyfunctional activity, including cytotoxic activity, the ability to proliferate, and the ability to produce the effector cytokines. The finding of the negative correlation between HTNV-NP FA9 epitope-primed CD8^+^ T-cell responses and disease severity suggests at least partial control of HTNV infection in HFRS patients (21).

Although the genome of the virus could potentially encode a multitude of T-cell epitopes, the specific T-cell response against viral infections often focuses on a few epitopes, which is known as immunodominance. The HLA-A*02-restricted HTNV-NP epitope FA9 that we previously identified was not only the immunodominant epitope but also a highly conserved epitope with 67–100% concordance among hantaviruses compared to the sequences of with other serotypes (21). To study the response induced by HLA-A*02-restricted HTNV-NP epitopes, we have previously used peptide–HLA (pHLA)-pentamers to detect the frequencies of HTNV-NP epitope-specific CD8^+^ T cells in the peripheral blood mononuclear cells (PBMCs) of HLA-A*02-positive HFRS patients (21). Because encounters between T-cell receptors (TCRs) and pHLA appear to be a key factor for the immunogenic potential of CD8^+^ T-cell responses, structural studies examining the pHLA should be highly relevant in understanding the cellular immune response to HTNV infection. X-ray crystal structures could reveal the structure of the closed conformation of the peptide/HLA complex (23–26). However, very few crystal structures demonstrating the interaction between HTNV epitopes and HLA molecules have been analyzed to date.

HLA-A*02 is the most prevalent HLA class I allele among the Chinese Han population, which has been considered to be associated with the control of many viral infections (27, 28). It is well-established that the HLA-A*02-restricted HTNV-NP FA9 epitope elicits highly effective CD8^+^ T-cell responses associated with reduced serum virus loads in HFRS individuals. However, it remains unclear whether the HTNV-NP FA9 epitope can induce a strong CD8^+^ T-cell immunity against HTNV infection *in vivo*. Humanized mice, which are mice engrafted with human tissue and/or are engineered to express human genes, are considered powerful tools for studying species-restricted pathogen that can induce *in vivo* human immunity (29–31). Therefore, a “humanized” HLA-A2.1/K^b^ transgenic (Tg) mouse model that expresses a chimeric monochain of HLA-A*02 infected with HTNV would be an ideal virus replication model to explore the effect of *in vivo* responses primed by epitope FA9. The study of HTNV-NP FA9 peptide-specific responses in HLA-A2.1/K^b^ Tg mice may provide an opportunity to further understand the *in vivo* mechanism of HTNV-specific CD8^+^ T cells against virus replication.

For immuno-prophylaxis of HTNV infection, inactive HTNV vaccines have been provided for free vaccinations against HFRS to high-risk populations in China for more than a decade. An epidemiological survey revealed the feasibility, safety, and immunogenicity of the inactive vaccine. However, the efficacy of the vaccine should be improved to further reduce the risk of infection (32). The peptides *in vivo* may mimic the epitopes that are presented and restricted the MHC to target cells, thus inducing relevant immune responses. Therefore, the development of peptide vaccines may be a better method to specifically direct the cellular immune system against pathogens. Given to the critical roles of the HTNV-NP FA9 epitope, which can induce protective CD8^+^ T-cell responses, such epitopes may be appealing targets for peptide vaccine development. However, although the HTNV-NP FA9 peptide presented by HLA-A*02 has been well characterized *in vitro*, the pHLA interaction, which may directly correlate to the efficacy of peptide vaccines and the effect of the HTNV-NP FA9 epitope in mice after challenge with HTNV remain largely unclear. Identifying these mechanisms is critical for the design of more effective HTNV peptide vaccines.

The collective results in this study illustrate the interaction between HTNV-NP peptide FA9 and HLA-A*02 molecules and demonstrate that FA9 vaccinations in HLA-A2.1/K^b^ Tg mice were able to control HTNV challenge upon induction of the effective cellular immune response to HTNV. The HTNV-NP FA9 peptide was identified as an immunogenic epitope that is naturally processed *in vivo* in an HLA-A*02-restricted manner. These findings provide crucial information for better evaluating T-cell immunity against HTNV infection, which may have important implications for the diagnosis and immuno-targeting of HFRS and may help guide the development of safe and effective T-cell-based HTNV peptide vaccines for humans.

## Materials and Methods

### Ethic Statement

This study was performed in strict accordance with the recommendations in the Guide for the Care and Use of Laboratory Animals of the National Health and Medical Research Council of China. The protocol was approved by the Committee on the Ethics of Animal Experiments of the Fourth Military Medical University with the license number XJYYLL-2014437. All procedures were performed under sodium pentobarbital anesthesia, and every effort was made to minimize animal suffering.

### Viruses and Vaccine

The HTNV 76-118 strain was kindly provided by the Department of Microbiology of our university. The commercial HFRS inactivated vaccine (YOUERJIAN^®^, Zhejiang Tianyuan Bio- Pharmaceutical Co., Ltd., China) derived from a mixture of both HTNV and SEOV was provided as a bivalent and purified HFRS vaccine.

### Peptide Synthesis

Based on the epitopes we identified previously (21), the 9-mer aa129–aa137 peptide on HTNV-NP (FVVPILLKA, FA9) restricted by HLA-A*02 and the aa131–aa139 peptide on HTNV-NP (VPILLKALY, VY9) restricted by HLA-B*35 were synthesized at 90% purity, as assessed by high-performance liquid chromatography and mass spectroscopy (CL Bio-scientific, Xi’an, China). Peptides were resuspended in a sterile phosphate-buffered saline (PBS) solution at 1 mM and stored in aliquots at −80°C before use.

### T2 Cells Binding Assay with Synthetic Peptides

A lymphoblast cell line, designated as T2 (174 × CEM. T2), was purchased from the American Type Culture Collection (cat. no. CRL-1992™; Manassas, VA, USA). This cell line is transporter-associated with antigen processing (TAP)-deficient and expresses HLA-A*02. Cells were cultured in RPMI 1640 culture medium containing 10% fetal bovine serum (Gibco, Grand Island, NY, USA), 100 U/ml penicillin and 100 mg/ml streptomycin. The T2 cell-binding assay was performed as described elsewhere to determine the binding capability of synthetic peptides to HLA-A*0201 molecules (33). Briefly, T2 cells were incubated with 50 μmol/l peptide, and 1 μmol/l human β2-microglobulin (β2m, Sigma) in serum-free RPMI 1640 medium for 18 h at 37°C with 5% CO_2_. The expression of HLA-A*0201 by the T2 cells was then determined by staining with fluorescein isothiocyanate (FITC)-labeled anti-HLA-A2 monoclonal antibody (mAb) BB7.2 (Biolegend) and detected by flow cytometry (FACScan; BD Biosciences). The results are presented as the fluorescent index (FI), which was determined as follows: FI = (mean PE fluorescence with the given peptide − mean PE fluorescence without peptide)/(mean PE fluorescence without peptide). FI ≥ 1 represents high-affinity peptides (34), indicating that the peptide stably combined with HLA-A*0201 molecules on the surface of T2 cells to increase the mean fluorescence of the HLA-A*0201 molecules by at least onefold.

### Protein Expression, Refolding, and Purification

Refolding of the HTNV-NP-derived FA9 peptide with the HLA-A*0201 heavy chain and β2m was performed as described previously (33–35). Briefly, the HLA-A*0201 heavy chain (extracellular domain aa1–aa275) and β2m were expressed as inclusion bodies in *Escherichia coli* using the pET prokaryotic expression system (Novagen Inc.). Then, the inclusion bodies were separately dissolved in a solution of 10 mM Tris/HCl (pH 8.0) and 8 M urea. The HLA-A*0201 heavy chain, β2m and the peptide were subsequently mixed at a molecular ratio of 1:1:3 into a refolding buffer (100 mM Tris/HCl, 400 mM l-arginine-HCl, 2 mM EDTA, 0.5 mM oxidized glutathione, and 5 mM reduced glutathione; Sigma). After incubation at 4°C for 24 h, the soluble refolded complex was concentrated using a bicinchoninic acid (BCA) protein assay (Pierce) and purified by gel filtration, followed by anion exchange chromatography using a Superdex G-200 (Amersham Pharmacia Biotech) to ensure the correctly refolded pHLA complex. Finally, the complex was concentrated to 10 mg/ml in 20 mM Tris–HCl (pH 8) and 50 mM NaCl for crystallization.

### X-Ray Crystallography, Data Collection, and Structure Determination

Crystal screens were initiated using commercial kits (Hampton Research). The crystals were obtained from vapor-diffusion hanging drops containing equal volumes of protein [10 mg/ml; 25 mM 2-(*N*-morpholino) ethanesulfonic acid (MES), pH 6.5] and 16% polyethylene glycol 6000 in 25 mM MES (pH 6.5). Plates were incubated at 291 K and examined after 72 h and 1 week. The diffractable crystals were soaked in a reservoir solution supplemented with 20% glycerol as a cryoprotectant, then flash-cooled and maintained at 100 K in a cryostream and obtained by subsequent microseeding. The data were collected on a Rigaku R-AXIS IV++ image plate using a Rigaku MicroMax 007 rotation-anode X-ray in-house generator (Rigaku) operating at 40 kV and 20 mA (Cu Kα; λ = 1.5418 Å). The data were processed and scaled using the HKL2000 (HKL Research Inc.).

### HLA-A2.1/K^b^ Transgenic Mice

HLA-A2.1/K^b^ Tg mice purchased from the Jackson Laboratory (BarHarbor, ME, USA) were kindly provided by Dr. Yuzhang Wu (Third Military Medical University, Chongqing, China) and maintained in the Animal House Facility at the Center for Laboratory Animal, Fourth Military Medical University, Xi’an, China. The Tg mice created in a C57BL/6 background represent a chimeric gene consisting of the α1 and α2 domains of HLA-A*0201 and the α3 transmembrane and cytoplasmic domain of H-2K^b^. Cell-surface HLA-A*02 expression was assessed by flow cytometry using phycoerythrin (PE)-labeled anti-HLA-A*02 mAb BB7.2.

### Immunization of HLA-A2.1/K^b^ Tg Mice with the Peptide

The HLA-A2.1/K^b^ Tg mice were divided into four groups (*n* = 6 each). Immunizations of the Tg mice with the peptide were carried out using the N-terminal fragment of the murine glycoprotein 96 (gp96) as an adjuvant (35, 36). Briefly, each 6- to 8-week-old male HLA-A2.1/K^b^ Tg mouse was immunized via subcutaneously injection at multiple sites with 50 μg of HLA-A*02-restricted FA9 peptide, emulsified in complete or incomplete Freund’s adjuvant (Difco) and 30 μg of the N-terminal fragment N333 (aa22–aa355) of murine gp96. The injection volume was adjusted to 100 μl for each animal. Three immunization injections were administered to each mouse at intervals of 2 weeks. The same method was used for immunization with the HLA-B*35-restricted epitope VY9 as the uncorrelated peptide control group. Subcutaneous injections with the HFRS inactivated vaccine and PBS were used as the positive and negative control groups, respectively. Ten days after the last immunization, the splenocytes were isolated and erylysed. Specific cytokine-producing cells were detected via an Enzyme-Linked Immunospot (ELISPOT) assay and intracellular staining.

### HTNV Challenge

A preliminary experiment was conducted to determine the infection of the HTNV 76-118 strain in naive HLA-A2.1/K^b^ Tg mice following HTNV challenge. Ten days after the final immunization booster, the mice were challenged with the HTNV 76-118 strain by intramuscular injection (1 × 10^5^ pfu/mouse). The Tg mice were sacrificed 4 days following HTNV challenge. Tissue samples, including the cerebrum, heart, liver, spleen, lungs, and kidneys, were weighed and prepared as 10% (gram/milliliter) tissue suspensions in PBS. Then, the tissue samples were freeze-thawed (−80°C/37°C) three times after grinding and centrifuged at 12,000 × rpm for 30 min at 4°C to collect the supernatants of the tissue.

### *Ex Vivo* IFN-γ Enzyme-Linked Immunospot Assay

The determination of the immunogenicity of the HTNV-NP 9-mer peptide *in vivo* was performed using an interferon (IFN)-γ ELISPOT assay (Mabtech, Büro Deutschland, Germany), as previously described (20). Briefly, the splenocytes obtained from HLA-A2.1/K^b^ Tg mice were placed in duplicate on ELISPOT plates at 1 × 10^6^ cells/well and stimulated overnight with peptides (10 μM). Cells with phytohemagglutinin (PHA, 10 μg/ml, Sigma-Aldrich, St. Louis, MO, USA) or no peptide stimulation served as positive and background controls, respectively. Spots formed by the deposition of the enzyme substrate were counted using an ELISPOT plate reader (Cellular Technology Limited, USA). Adjusted spot-forming cells (SFC) after subtracting the average negative values are expressed as SFC/10^6^ splenocytes. The positive response was defined as at least 50 SFC/10^6^ input cells, exceeding three times the background response after subtracting the number of spots in the background controls from those in the stimulated samples. The SFC/10^6^ splenocytes in the unstimulated control wells never exceeded five spots per well.

### Intracellular Cytokine Staining and CD107a Degranulation Assay

Splenocytes (2 × 10^6^) obtained from HLA-A2.1/K^b^ Tg mice that had been immunized were restimulated with peptide (5 μM), as previously described (20). Briefly, Brefeldin A (10 μg/ml; Sigma-Aldrich, St. Louis, MO) and the costimulatory molecules anti-CD49d (clone 9F10) and anti-CD28 (clone CD28.2) (1 μg/ml, Biolegend) were added to the culture for 6 h at 37°C. The CD107a-PE antibody (BD Pharmingen) was added to the wells during the stimulation. Cells stimulated with phorbol myristate acetate (PMA, 0.1 μg/ml, Sigma-Aldrich, St. Louis, MO, USA)-ionomycin (0.05 μg/ml, Sigma-Aldrich, St. Louis, MO, USA) or medium alone were used as positive and negative controls, respectively. Cells were labeled with anti-mouse CD3 and anti-mouse CD8α mAbs (BD Biosciences Pharmingen), fixed and permeabilized using a BD Cytofix/Cytoperm kit (BD Biosciences Pharmingen), and then stained with PE or APC-labeled anti-IFN-γ, PE-labeled anti-tumor necrosis factor (TNF)-α, FITC-labeled anti-granzyme B and PE-labeled anti-CD107a mAbs (BD Biosciences Pharmingen). FITC-, PE-, PerCP–Cy5.5-, and APC-conjugated mouse IgG1, κ were used as isotype controls of the 4-color staining. A total of 200,000 events per sample were collected using a FACSCalibur flow cytometer (BD Biosciences). The analysis was performed immediately with FlowJo version 9.2 (TreeStar). Splenocytes were defined as FSC/SSC, and CD8^+^ T cells were defined as CD3^+^CD8^+^ events, displayed on a dot plot of CD8 versus cytokines. The cytokine response was considered positive when the percentage of cytokine was greater than 0.1% after background subtraction.

### Detection of HTNV Antigens by Enzyme-Linked Immunosorbent Assay

Hantaan virus antigens in the supernatants of the major tissues of Tg mice were detected by sandwich enzyme-linked immunosorbent assay (ELISA). Anti-HTNV-NP mAb 1A8 was prepared in the Department of Microbiology of our university. The mAb 1A8 and horseradish peroxidase-conjugated 1A8 were used as the coating and detecting antibodies, respectively (37). The normal tissue supernatants were used as negative controls. The absorbance was measured at a wavelength of 490 nm using a standard ELISA plate reader (Bio-red). OD490 values exceeding 2.1-fold of the negative controls (P/N) were considered positive results.

### Determination of Relative HTNV RNA Loads with Quantitative RT-PCR

The major organs, including the heart, liver, spleen, lungs, kidneys, and brain, of post-exposure Tg mice were preserved in a non-frozen tissue RNA preservation solution (Solarbio, CN) at −4°C. The tissue RNA of each organ was extracted using RNA purification extraction kits (Tiangen Biotech, CN) and used as a template for reverse transcription using PrimeScript™ RT-PCR kit (Takara) to obtain the cDNA. The target sequence of the HTNV S segment was detected in the major organs using a SYBR real-time quantification PCR kit (Takara) with the following primers: HTNV forward, 5′-GATCAGTCACAGTCTAGTCA-3′; HTNV reverse, 5′-TGATTCTTCCACCATTTTGT-3′; mouse GAPDH forward, 5′-AGGCCGGTGCTGAGTATGTC-3′; mouse GAPDH reverse, 5′-TGCCTGCTTCACCACCTTCT-3′. The results were recorded as cycle time (*Ct*) and quantified by 2^−ΔΔ^*^Ct^*.

### Statistical Analysis

Statistical analyses and graphing were performed using SPSS 16.0 (SPSS Inc., Chicago, IL, USA) and Prism software, version 5.0 (Graphpad; La Jolla, CA, USA). The frequency of the CD8^+^ T cells and the cytokines secreted are presented as the medians and range values. The Wilcoxon rank-sum test was used for parameter comparison between the two subject groups. A two-tailed *P*-value below 0.05 (*p* ≤ 0.05) was considered statistically significant.

### Accession Numbers

HTNV 76-118 strain nucleoprotein GenBank accession number: M14626. The hantaviruses used in this study, corresponding to the HTNV 76-118 strain: NC_005218. The structure of the GV9 (GLMWLSYFV)/HLA-A*0201complex was deposited in the Protein Data Bank with the accession number: PDB code 3I6G.

## Results

### The Synthesized HTNV-NP FA9 Peptide Exhibited High Binding Affinity to the HLA-A*0201 Molecule

**Figure 1:**
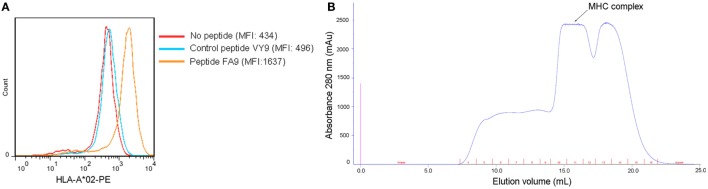
Binding affinity of the HTNV nucleoprotein FA9 peptide to HLA-A*0201 molecule. **(A)** MHC stabilization assay with T2 cells was used to quantify the peptide-binding affinity of HTNV nucleoprotein (NP) FA9 (aa129–aa137, FVVPILLKA) to HLA-A*0201 molecule via flow cytometry. HLA-B*35-restricted HTNV-NP VY9 (aa131–aa139, VPILLKALY) unrelated peptides served as negative controls. The red curve indicates T2 cells incubated without peptide. The blue curve indicates T2 cells incubated with peptide VY9. The orange curve indicates T2 cells incubated with peptide FA9. The overlay of the three conditions in histograms clearly demonstrate that the curve of T2 cells incubated with peptide FA9 is shifted more to the right than that incubated with peptide VY9 or without peptide. The results shown are representative of three independent experiments. **(B)** *In vitro* refolding of the HTNV-NP FA9 peptide with the HLA-A*0201 heavy chain and β2m. The refolded complex was analyzed by gel filtration chromatography using a Superdex 200 16/60 column. The peak of the HLA complex as indicated by the arrow with the expected molecular mass of 45 kDa eluted at the estimated volume of 15.9 ml. mAU, milli-absorbance units.

Our previous findings showed that the HTNV-NP FA9 peptide restricted by HLA-A*02 induced an effective CD8^+^ T-cell response in HFRS patients (21). To further investigate the binding affinity of peptide FA9 to an HLA-A*02 molecule, a T2 cell-peptide-binding assay was conducted. The synthesized peptide FA9 increased the surface expression of HLA-A*0201 molecules, corresponding to a FI value of 2.77, indicating that peptide FA9 had a high affinity for binding with HLA-A*0201 molecules, whereas the HLA-B*35-restricted peptide aa131–aa139 (VPILLKALY, VY9) on HTNV-NP used as a negative control showed an FI of 0.14, indicating no binding with HLA-A*0201 (Figure 1A). Moreover, the HLA-A*0201 complex refolding assay showed that the predicted molecular weight of the protein coming out with ~15.9 ml eluting buffer would be 45 kDa, coincident with the molecular mass of the pHLA complex (Figure 1B and Figure S1 in Supplementary Material). Therefore, the results suggest that the HTNV-NP FA9 peptide binds with an HLA-A*0201 molecule with a high binding affinity.

### Structural Evidence of Peptide Presentation of HTNV-NP FA9 by the HLA-A*0201 Molecule

**Figure 2:**
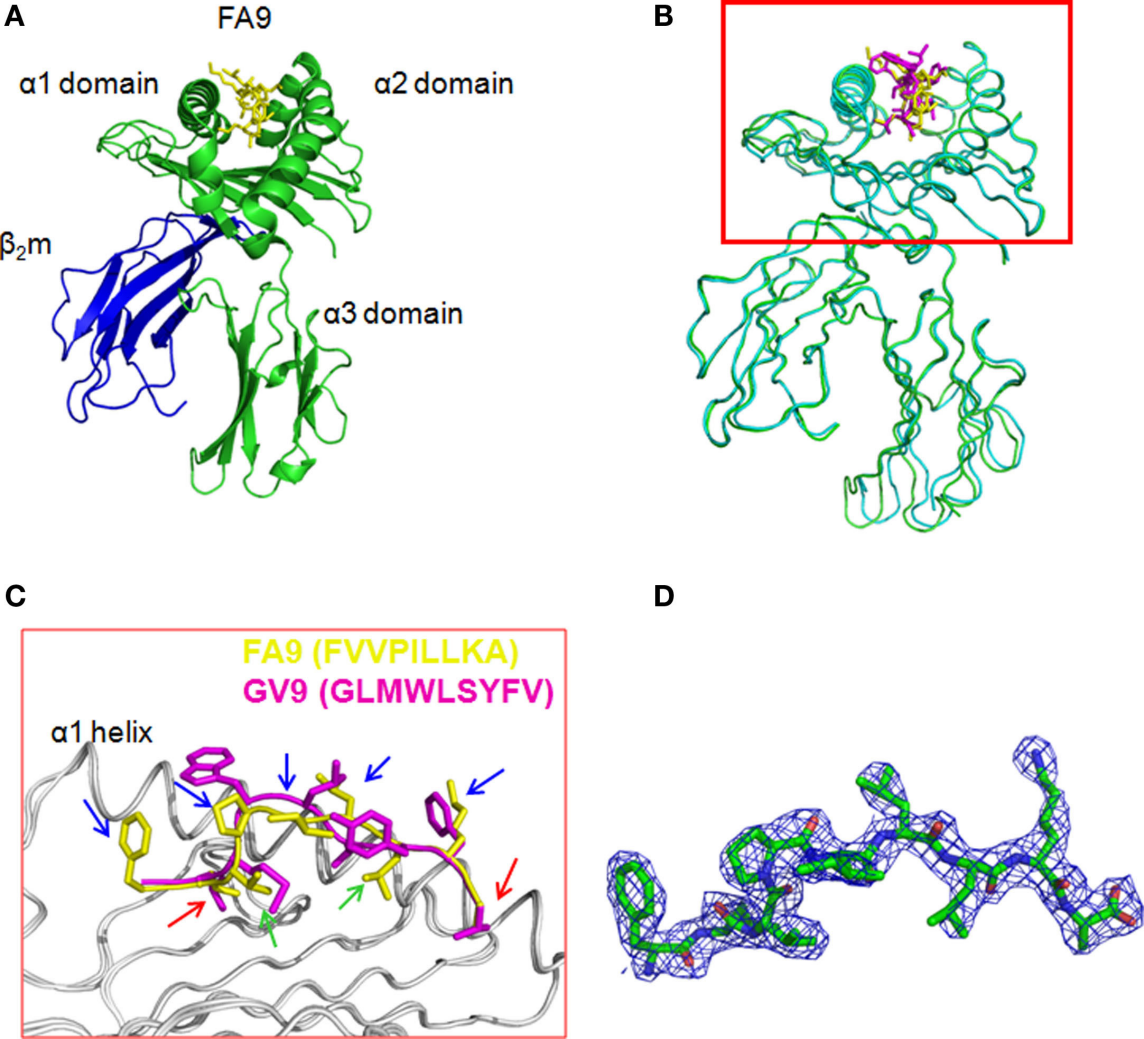
The structure of HLA-A*0201 complexed with the HTNV nucleoprotein FA9 peptide. The crystals were obtained from vapor-diffusion hanging drops and the data were processed and scaled using the HKL2000. **(A)** Overview of the three-dimensional structure of the FA9/HLA-A*0201complex showing a typical MHC class I fold. The antigenic peptide-binding groove was constituted by antiparallel α1 and α2 helices in the extracellular region of the HLA-A*0201 heavy chain, and supported by an eight-stranded β-sheet bedplate. The HTNV nucleoprotein (NP) FA9 (aa129–aa137, FVVPILLKA) peptide was presented in the peptide-binding cleft. The α3 domain and β2m occupied the standard positions below the bedplate. **(B)** Structural superposition of the FA9/HLA-A*0201 complex and the previously determined HLA-A*0201 structure complexed with peptide GV9 (GLMWLSYFV, PDB code: 3I6G). The structure of FA9/HLA-A*0201 is similar to the structure of GV9/HLA-A*0201 with a low root mean square difference (RMSD) of 0.415 Å. **(C)** The alignment of peptide FA9 with peptide GV9. The mainchain conformation of FA9 is similar to GV9. The residues Val2 and Ala9 (red arrows) act as the primary anchor residues of FA9, which exhibit the same conformations as Leu2 and Val9 of GV9. The residues Val3 and Leu7 (green arrows) act as secondary anchor residues for peptide FA9, whereas the residues Met3 and Ser6 might be the secondary anchors for the peptide GV9. The residues Phe1, Pro4, Ile5, Leu6, and Lys8 (blue arrows) of peptide FA9 pointing upwards from the HLA-A*0201 surface are the most probable recognition sites for TCRs. **(D)** The electron density for peptide FA9 at σ = 1.0 contour level.

To further confirm that the HTNV-NP FA9 peptide is a typical HLA-A*02-restricted epitope, the crystal structure of peptide FA9 bound to HLA-A*0201 was further analyzed. Crystal trials were carried out with the purified heterodimer of the heavy chain and β2-microglobulin (β2m) expressed in *E. coli*, and the synthesized cognate peptide was refolded. The diffracting crystals of pHLA were then obtained (Figure S2 in Supplementary Material). As expected, the overall three-dimensional structure and domain arrangements of the FA9/HLA-A*0201 complex showed a typical MHC class I fold, which was exactly similar to those of the formerly determined peptide/HLA-A*0201 molecules (Figure 2A). Specifically, the antiparallel α1 and α2 helices in the extracellular region of the HLA-A*0201 heavy chain formed the antigenic peptide-binding groove, which was supported by an eight-stranded β-sheet bedplate. The α3 domain and β2m occupied the standard positions below the bedplate (Figure 2A). Compared with the previously determined HLA-A*0201 complexed with peptide GV9 (GLMWLSYFV, PDB code: 3I6G), the structural superposition of FA9/HLA-A*0201 was similar to that of GV9/HLA-A*0201 with a low root mean square difference (RMSD) of 0.415 Å. The amino acid sequence alignments revealed that the main chain conformation of FA9 is quite similar to GV9 (Figures 2B,C). Specifically, the residues Val2 and Ala9 act as primary anchor residues of peptide FA9, which perform the same conformation as Leu2 and Val9 of peptide GV9. The residue Val at position 2 from the N-terminus and the residue Ala9 at the C-terminal of peptide FA9 were deeply buried in the pocket, indicating typical anchor residues (Figure 2C). Of particular interest were the residues Val3 and Leu7, which point their side chains into the pocket and might act as secondary anchor residues for peptide FA9 to bind with HLA-A*0201. However, Met3 and Ser6 might be the secondary anchors for the peptide GV9 (Figure 2C). Although there were differences in residues observed between the FA9 and GV9 peptides, the crystal structures revealed that the two peptides adopted similar conformations in their binding with an HLA-A*0201 molecule. All of these anchor residues help the peptide tightly bind with the HLA-A*0201 heavy chain and enhance the stability of the entire complex. It is also important to note that the centrally located residues on the main chain of peptide FA9 tended to protrude out of the antigen-binding groove. The side chains of Phe1, Pro4, Ile5, Leu6, and Lys8 are the most exposed residues pointing upwards from the HLA-A*0201 surface, indicating that these amino acids are the most probable recognition sites for TCRs and might be involved in TCR docking (Figure 2C). The mutants in these positions may influence the antigenicity of the peptide and even lead to the immune escape of the virus. The further unambiguous electron density of the complex was observed for the bound peptide FA9, which was well defined inside the peptide-binding groove of HLA-A*0201 (Figure 2D). The structural properties of the FA9/HLA-A*0201 complex may be related to the immunogenicity of the epitope that allow it to induce specific immune response to against HTNV infection.

### *In vivo* Induction of Peptide-Specific CD8^**+**^ T Cells in HLA-A2.1/K^b^ Tg Mice Immunized with the HTNV-NP FA9 Peptide

**Figure 3:**
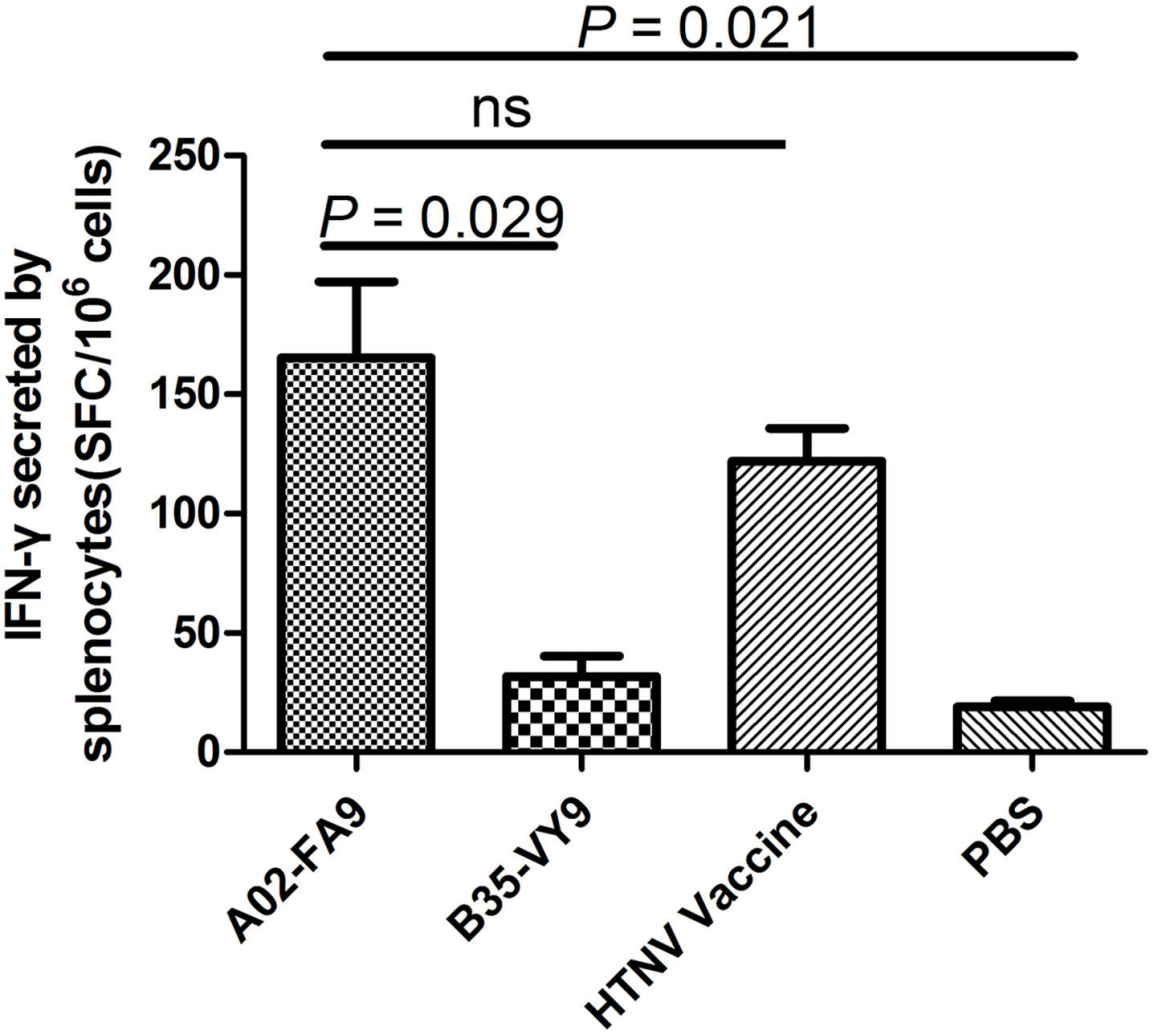
**The analysis of interferon (IFN)-γ secretion by splenocytes of HLA-A2.1/Kb transgenic mice against HTNV nucleoprotein peptide FA9**. Comparison of the magnitudes (*y-axis*) of *ex vivo* ELISPOT IFN-γ responses to the HTNV nucleoprotein (NP) FA9 peptide (aa129–aa137, FVVPILLKA) among the different groups (*x-axis*). The HLA-A2.1/K^b^ transgenic mice were divided into four groups (*n* = 6 each), including the mice immunized with the HLA-A*02-restricted HTNV-NP FA9 peptide, the HLA-B*35-restricted HTNV-NP VY9 (aa131–aa139, VPILLKALY) as an unrelated peptide control, the HTNV vaccine as a positive control and PBS as a negative control, respectively. Three immunization injections were administered to each mouse at intervals of 2 weeks. Ten days after the last immunization, the splenocytes were isolated for IFN-γ detection by ELISPOT assay. The magnitudes of the responses are represented as the spot-forming cells (SFC)/10^6^ splenocytes. A02-FA9 indicates the HLA-A2.1/K^b^ transgenic mice immunized with the HLA-A*02-restricted HTNV-NP FA9 peptide. B35-VY9 indicates the transgenic mice immunized with the HLA-B*35-restricted HTNV-NP VY9 peptide. The Wilcoxon rank-sum test was used for statistical evaluation. ns, not significant.

Having defined the structure of the complex HTNV-NP FA9 peptide/HLA-A*0201 molecule, we next sought to determine the *in vivo* immunogenic potential of peptide FA9, which was validated for its ability to elicit specific CD8^+^ T-cell responses *in vitro* in our previous study. Groups of HLA-A2.1/K^b^ Tg mice were immunized or injected with peptide HTNV-NP FA9, peptide HTNV-NP VY9, the HTNV inactivated vaccine, or PBS, as described in Section “Materials and Methods.” After three rounds of *in vivo* stimulation, splenocytes from primed mice were first tested for IFN-γ production via an ELISPOT assay with peptide FA9 as the stimulator. The results showed that a large number of CD8^+^ T cells from Tg mice inoculated with peptide FA9 demonstrated strong IFN-γ production with a median magnitude of 151 (range, 104–255) SFC/10^6^ splenocytes, which is even higher than the IFN-γ secretion level detected in the positive control group injected with the HTNV inactivated vaccine (median: 123, range: 89–152 SFC/10^6^ splenocytes), although the difference was not statistically significant. By contrast, no specific CD8^+^ T-cell reactivity was detected in the splenocytes generated from Tg mice injected with PBS (median: 18, range: 14–26 SFC/10^6^ splenocytes) (Figure 3). More importantly, although immunization with the FA9 peptide in Tg mice efficiently elicited FA9-specific CD8^+^ T-cell responses, the HLA-B*35-restricted HTNV-NP VY9 peptide used as an uncorrelated peptide control was unable to prime or very inefficiently primed obvious CD8^+^ T-cell responses in the Tg mice, with a median value of 28 (range, 15–55) SFC/10^6^ splenocytes for IFN-γ production (Figure 3). Notably, compared to Tg mice injected with PBS, the SFC count, as an index of the CD8^+^ T-cell reactivity, increased by approximately eightfold in the FA9 peptide-immunized mice (*p* = 0.021) (Figure 3). Moreover, a comparison of IFN-γ secretion between the FA9 peptide group and the uncorrelated VY9 peptide control group revealed a significant quantitative difference in the magnitude of peptide FA9-specific CD8^+^ T-cell responses. Immunization with the FA9 peptide elicited significantly stronger IFN-γ producing CD8^+^ T-cell responses than that observed in Tg mice inoculation with the VY9 peptide (*p* = 0.029) (Figure 3). These results indicate that HTNV-NP FA9 peptide can be naturally processed *in vivo* in an HLA-A*02-restricted manner and induce a robust FA9-specific CD8^+^ T-cell response in HLA-A2.1/K^b^ Tg mice.

### HTNV-NP FA9 Peptide Elicited High Levels of Cytokine and Cytotoxic Mediator Production in HLA-A2.1/K^b^ Tg Mice

**Figure 4:**
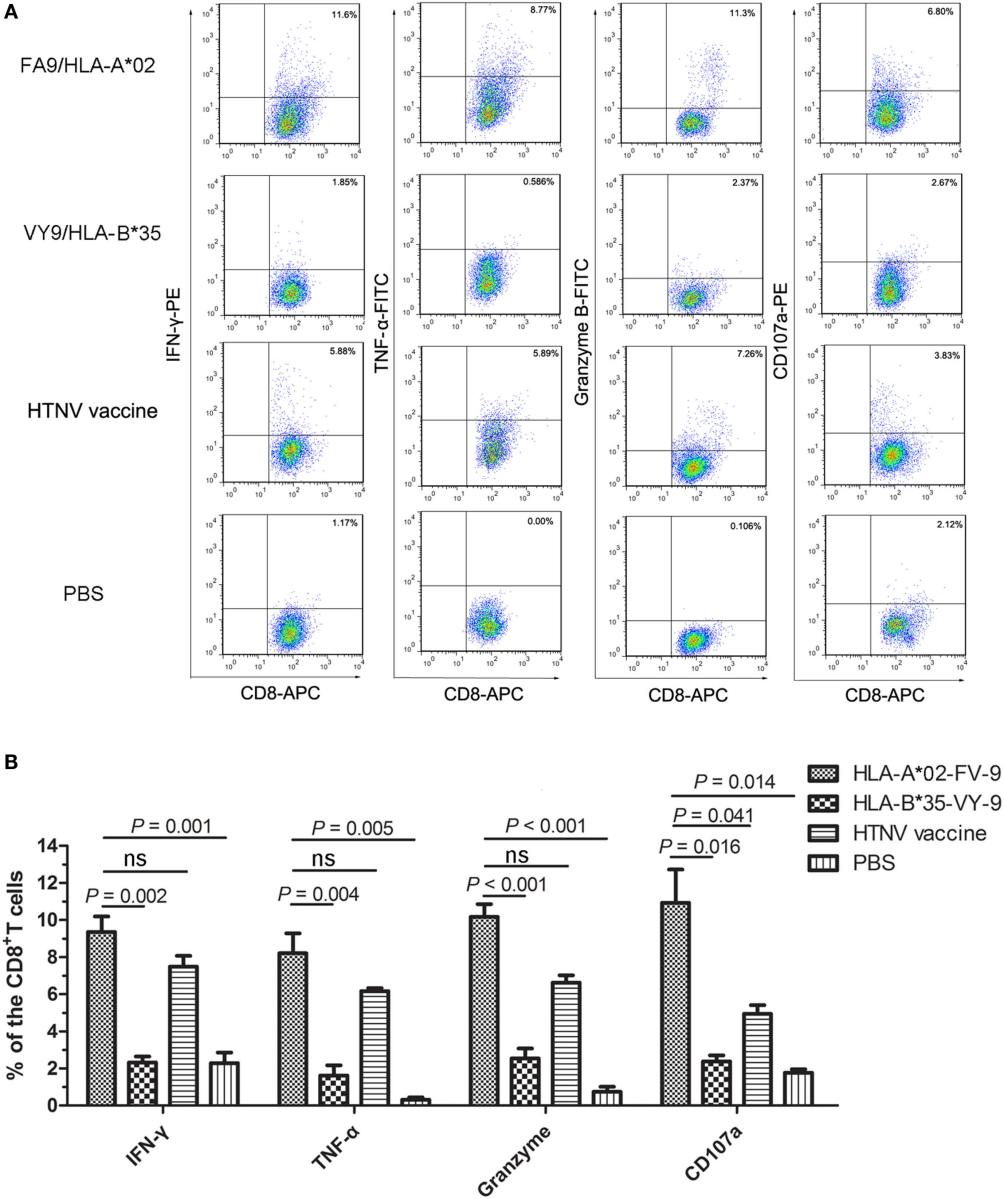
**The cytokine and cytolytic mediator production of splenocytes in HLA-A2.1/K^b^ transgenic mice after peptide immunization**. The HLA-A2.1/K^b^ transgenic mice were divided into four groups (*n* = 6 each) according to the different immunization, including the HLA-A*02-restricted HTNV-NP FA9 peptide (aa129–aa137, FVVPILLKA), the HLA-B*35-restricted HTNV-NP VY9 peptide (aa131–aa139, VPILLKALY), the HTNV vaccine and PBS immunized mice groups, respectively. **(A)** Representative flow cytometric plots of the cytokines interferon (IFN)-γ and tumor necrosis factor (TNF)-α or cytotoxic mediator granzyme B-producing and CD107a-expressing CD8^+^ T cells in splenocytes (*vertical*) after three immunizations with the HLA-A*02-restricted HTNV-NP FA9 peptide, the HLA-B*35-restricted HTNV-NP VY9 peptide, the HTNV vaccine or PBS (*horizontal*). The numbers indicate the percentage of cells within the boxed regions. **(B)** Comparison of the frequencies (*y-axis*) of cytokines IFN-γ and TNF-α and cytotoxic mediator granzyme B and the expression percentage of CD107a (*x-axis*) in the peptide FA9-specific CD8^+^ T cells between the peptide FA9-immunized mice and control mice, including peptide VY9-immunized mice as an unrelated peptide control, HTNV-vaccinated mice as a positive control and PBS-administered mice as a negative control, respectively. The results shown are representative of three independent experiments. The Wilcoxon rank-sum test was used for statistical evaluation. ns, not significant.

To define the phenotype of FA9-responsive CD8^+^ T cells more precisely, splenocytes from each group of HLA-A2.1/K^b^ Tg mice were directly stimulated *ex vivo* with the FA9 peptide, and the number of CD8^+^ T cells showing specifically inducible cytokine secretion and cytotoxic mediator expression was assessed via intracellular staining analysis. The representative analyses of these distinct patterns of staining are shown in Figure 4A. Overall, CD8^+^ T cells in the splenocytes from Tg mice after immunization displayed cytokine or cytotoxic mediator production, predominantly characterized by IFN-γ, TNF-α, granzyme B secretion, and CD107a expression upon stimulation with peptide FA9. Consistent with the detection of IFN-γ in the ELISPOT assay, a marked high frequency of IFN-γ as the primary cytokine was induced in the splenocytes of Tg mice immunized with the FA9 peptide (median: 8.99%, range: 7.85–11.60% of CD8^+^ T cells). A high frequency of TNF-α-producing cells was also observed in peptide FA9-immunized mice (median: 7.74%, range: 6.34–11.00% of CD8^+^ T cells) (Figure 4B). Further analysis revealed that the CD8^+^ T cells displayed adequate cytolytic capacity and appeared to be highly functional, secreting cytolytic effector granzyme B (median: 10.45%, range: 8.48–11.30% of CD8^+^ T cells) and upregulating CD107a expression (median: 10.85%, range: 6.80–15.20% of CD8^+^ T cells) after stimulation with the FA9 peptide (Figure 4B). Interestingly, similar to what has been observed in *ex vivo* comparisons of IFN-γ production, CD8^+^ T cells from the FA9-immunized mice displayed a trend toward higher levels of cytokines production and cytolytic mediator expression than the secretion of CD8^+^ T cells from other control groups. As shown in Figure 4B, the Tg mice injected with PBS used as a negative control had few or no detectable cells that expressed cytokines or cytolytic mediators, and the frequencies of cells expressing cytokines or cytolytic mediators were all significantly lower than the frequencies induced in the FA9-immunized group (*p* = 0.001 for IFN-γ, *p* = 0.005 for TNF-α, *p* < 0.001 for granzyme B and *p* = 0.014 for CD107a). The percentages of IFN-γ (median: 7.67%, range: 5.88–8.70% of CD8^+^ T cells), TNF-α (median: 6.09%, range: 5.89–6.61% of CD8^+^ T cells), and granzyme B (median: 6.81%, range: 5.59–7.26% of CD8^+^ T cells)-producing CD8^+^ T cells in the positive control group inoculated with the HTNV inactivated vaccine were slightly lower than the frequencies of the FA9 stimulation group, although this difference was not significant. However, a higher frequency of CD107a expression was observed in the CD8^+^ T cells from Tg mice immunized with peptide FA9 than in those of mice immunized with the HTNV-inactivated vaccine (median: 4.99%, range: 3.83–5.95% of CD8^+^ T cells, *p* = 0.041). Importantly, compared with the substantially higher frequencies of cytokine secretion and cytolytic mediator expression by specific CD8^+^ T cells in the peptide FA9-immunized Tg mice, the Tg mice inoculated with the HLA-B*35-restricted VY9 peptide showed much less IFN-γ (median: 2.21%, range: 1.75–3.11% of CD8^+^ T cells, *p* = 0.002), TNF-α (median: 1.40%, range: 0.59–3.11% of CD8^+^ T cells, *p* = 0.004), and granzyme B (median: 2.79%, range: 1.08–3.50% of CD8^+^ T cells, *p* < 0.001) production and CD107a expression (median: 2.62%, range: 1.42–2.85% of CD8^+^ T cells, *p* = 0.016), further confirming the specificity of the HLA–peptide interaction for inducing effective T-cell responses. Given that granzyme B release is considered a quantitative indicator of the cytotoxic activity of T cells, these observations indicated that FA9-specific CTLs with significant upregulation of granzyme B and CD107a expression might possess the cytotoxic capacity to directly kill HTNV-infected cells.

### Immunization with the HTNV-NP FA9 Peptide Reduced HTNV Titers in Tissue Supernatants of HLA-A2.1/K^b^ Tg Mice after HTNV Challenge

Having demonstrated that HTNV-NP FA9 could elicit high levels of specific CD8^+^ T-cell responses in Tg mice, we then conducted HTNV challenge experiments to determine whether the peptide FA9-specific CD8^+^ T-cell responses could defend against HTNV infection *in vivo* and, thus, be used as a vaccine candidate. For this purpose, we established a model of HTNV infection based on replication of the virus *in vivo* in HLA-A2.1/K^b^ Tg mice. The Tg mice were grouped and immunized as before and subsequently challenged by the administration of the HTNV strain 10 days after immunization. Tissue samples from the cerebrum, heart, liver, spleen, lung, and kidneys of the Tg mice were harvested from each mouse on day 4 post-challenge and detected for HTNV titer.

**Figure 5:**
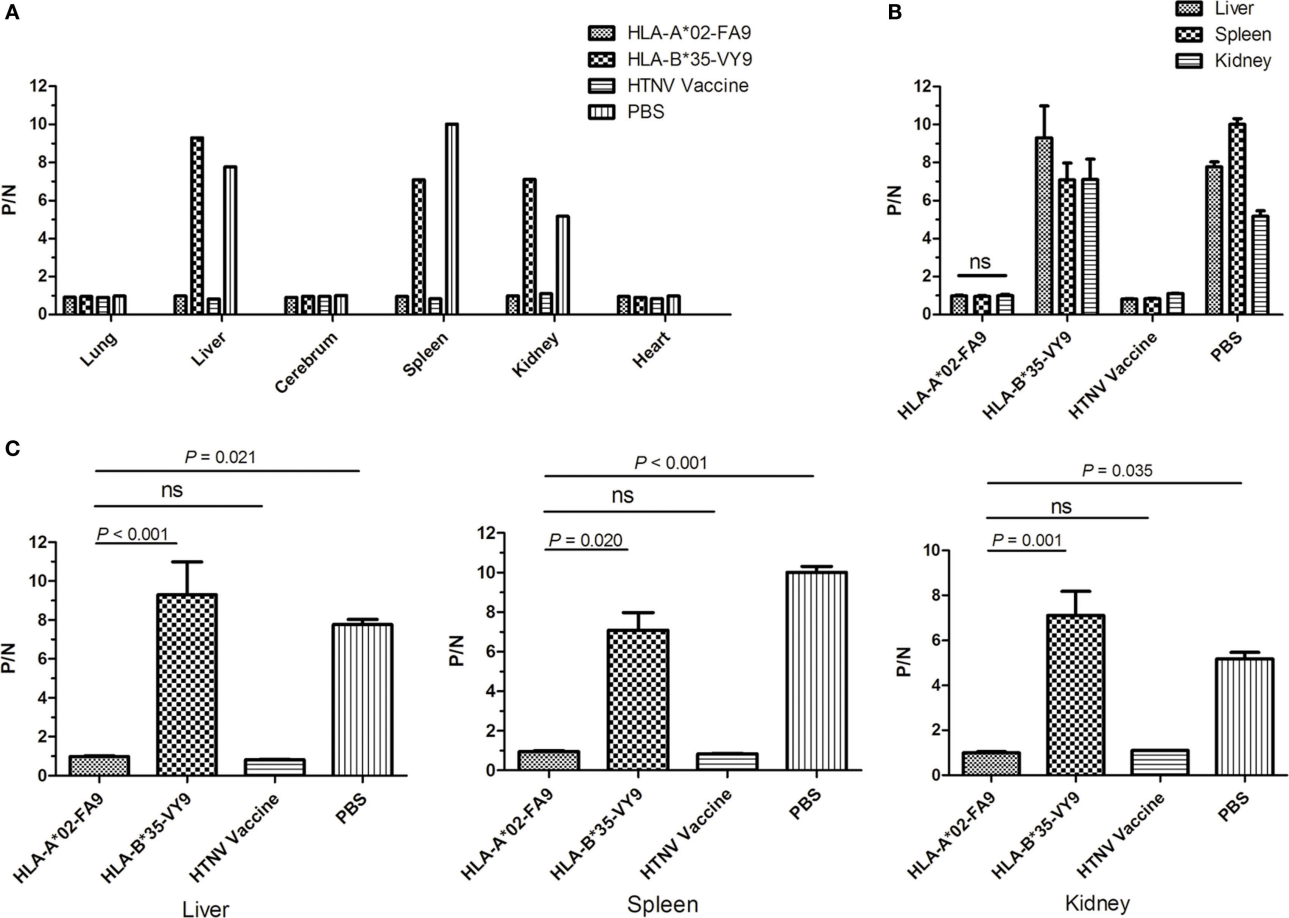
**Detection of HTNV antigen with ELISA in the organs of HLA-A2.1/K^b^ transgenic mice after HTNV challenge**. The HLA-A2.1/K^b^ transgenic mice were divided into four groups (*n* = 6 each) according to the different immunization, including the mice immunized with the HLA-A*02-restricted HTNV-NP peptide FA9 (aa129–aa137, FVVPILLKA), the HLA-B*35-restricted HTNV-NP peptide VY9 (aa131–aa139, VPILLKALY), the HTNV vaccine or PBS, respectively. Ten days after the final immunization booster, the mice were challenged with the HTNV 76-118 strain and sacrificed 4 days following HTNV challenge. **(A)** The HTNV antigen (*y-axis*) was detected in the lungs, liver, cerebrum, spleen, kidneys, and heart (*x-axis*) of HLA-A2.1/K^b^ transgenic mice after HTNV infection in the four groups, respectively. Few or no detectable virus was found in lungs, cerebrum, and heart in all four groups of immunized transgenic mice after HTNV challenge detected via ELISA. **(B)** Comparison of the HTNV antigen levels (*y-axis*) in the tissue supernatant of the liver, spleen, and kidneys in each immunized group (*x-axis*). **(C)** Comparison of the HTNV antigen levels (*y-axis*) between the HLA-A*02-restricted HTNV-NP peptide FA9immunized mice group and HLA-B*35-restricted HTNV-NP peptide VY9-immunized mice group or PBS-injected mice group (*x-axis*) in the organs liver, spleen and kidneys, respectively. The results shown are representative of three independent experiments. A P/N ratio >2.1 is considered positive. The Wilcoxon rank-sum test was used for statistical evaluation. ns, not significant.

The preliminary measurement of the viremia in six organs of the HTNV-infected naïve Tg mice showed that high levels of HTNV antigens could be detected in the liver, spleen, and kidneys, but not in lungs, cerebrum, and heart of the Tg mice, suggesting that the liver, spleen, and kidneys are the major organs for infection and replication of HTNV in HLA-A2.1/K^b^ Tg mice (Figure S3 in Supplementary Material). Consistent with the preliminary analyses, few or no detectable virus was found in lungs, cerebrum, and heart in all four groups of immunized Tg mice after HTNV challenge when HTNV antigens in the tissue supernatant were detected via ELISA (Figure 5A).

As expected, after HTNV challenge, each Tg mouse from the negative control group injected with PBS displayed evidence of high-titer HTNV replication in the tissue supernatant of the liver, spleen, and kidneys, whereas a significant reduction of the mean viral titer was observed in the organs of the mice immunized with the HTNV inactive vaccine (Figure 5B). Importantly, prior immunization with peptide FA9 led to a significant decrease in HTNV titers in the supernatants of the liver, spleen, and kidneys in mean viral titer compared to the negative control Tg mice (*p* = 0.021 for liver, *p* < 0.001 for spleen, and *p* = 0.035 for kidneys, Figure 5C). However, immunization with irrelevant control peptide HTNV-NP VY9 showed still high levels of HTNV titer in the supernatants of the liver, spleen, and kidneys in Tg mice, which was markedly higher than that in the FA9-immunized mice following HTNV challenge (*p* < 0.001 for liver, *p* = 0.020 for spleen, and *p* = 0.001 for kidneys, Figure 5C). Notably, although FA9 peptide immunization significant decreased the HTNV antigen titer in the Tg mice, there was no reduction in the levels found in the liver, spleen, and kidneys of the immunized Tg mice (Figure 5B), indicating the efficiency of CD8^+^ T-cell responses induced by peptide FA9 to comprehensively inhibit virus replication and clear the HTNV, therefore protecting against HTNV infection in HLA-A2.1/K^b^ Tg mice.

### Immunization with the HTNV-NP FA9 Peptide Inhibited HTNV Replication in HLA-A2.1/K^b^ Tg Mice to Protect against HTNV Challenge

**Figure 6:**
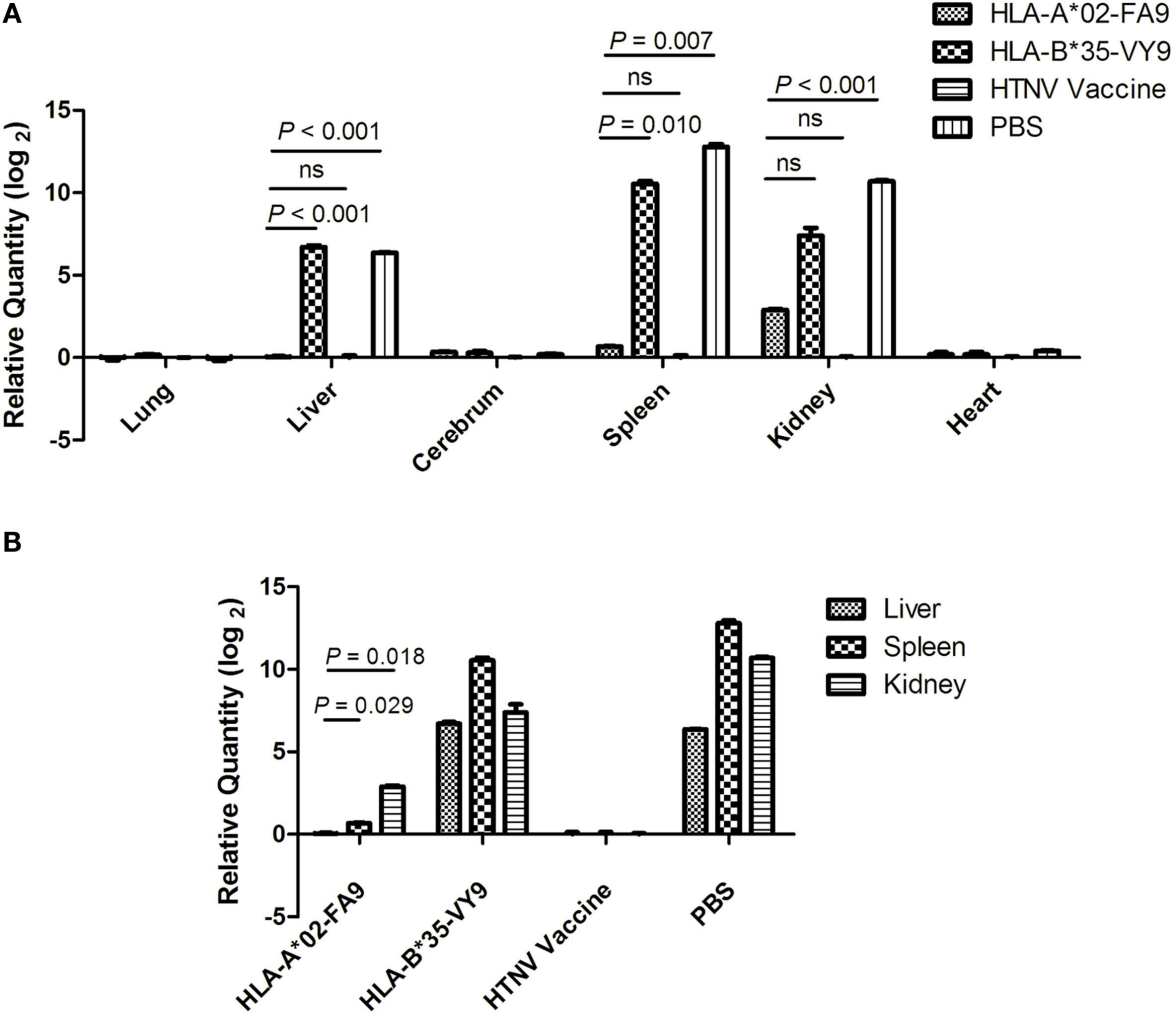
**Detection of HTNV RNA loads via real-time PCR in the organs of HTNV-challenged HLA-A2.1/K^b^ transgenic mice**. The HLA-A2.1/K^b^ transgenic mice were divided into four groups (*n* = 6 each), including the mice immunized with HLA-A*02-restricted HTNV-NP peptide FA9 (aa129–aa137, FVVPILLKA), HLA-B*35-restricted HTNV-NP peptide VY9 (aa131–aa139, VPILLKALY), HTNV vaccine or PBS, respectively. Ten days after the final immunization booster, the mice were challenged with the HTNV 76-118 strain and sacrificed 4 days following HTNV challenge. **(A)** The detection of HTNV RNA loads (*y-axis*) in the lungs, liver, cerebrum, spleen, kidneys, and heart (*x-axis*) of HLA-A2.1/K^b^ transgenic mice after HTNV challenge in the four groups. Comparison of the HTNV RNA loads that could be detected in liver, spleen, and kidneys of the immunized transgenic mice after HTNV challenge among four immunization mice groups. **(B)** The HTNV RNA loads (*y-axis*) in the liver, spleen, and kidneys of each immunized group (*x-axis*) and comparison of the HTNV RNA loads between the liver and spleen or between the liver and kidneys in the HLA-A*02-restricted HTNV-NP peptide FA9-immunized mice group. The results shown were recorded as cycle time (*Ct*) and quantified by 2^−ΔΔCt^ and were representative of three independent experiments. The Wilcoxon rank-sum test was used for statistical evaluation. ns, not significant.

The results showing that HTNV-NP FA9 peptide-specific CD8^+^ T-cell responses decreased HTNV titers in tissue supernatants of HTNV-infected Tg mice were confirmed following an HTNV RNA load analysis for the organs of Tg mice after HTNV challenge. As a more sensitive and accurate assay, real-time PCR specific for the HTNV S segment was carried out to determine the HTNV RNA loads isolated from the organs of the peptide-immunized mice challenged with HTNV. As shown in Figure 6A, the HTNV RNA in the lungs, cerebrum, and heart was not detectable by real-time PCR. However, we detected significant differences in the HTNV RNA loads between the Tg mice that received peptide FA9 inoculation and the mice immunized with the control peptide or injected with PBS. In accordance with the results of the HTNV antigen detection, significantly lower levels of HTNV RNA loads were observed in the liver, spleen, and kidneys of the Tg mice after immunization with the FA9 peptide than in those of the negative control mice (*p* < 0.001 for liver, *p* = 0.007 for spleen, and *p* < 0.001 for kidneys, Figure 6A). The mice immunized with the irrelevant control peptide HTNV-NP VY9 showed markedly higher levels of HTNV RNA loads in the liver and spleen than the FA9-immunized mice (*p* < 0.001 for liver and *p* = 0.010 for spleen, Figure 6A), whereas no significant difference was observed for HTNV RNA loads in the kidneys between the VY9 peptide-treated mice and the FA9-immunized mice (Figure 6A), although the inhibitory tendency of the HTNV RNA loads in the FA9 peptide immunization group was still observed in the kidneys. Notably, when the capacity of FA9 peptide immunization to reduce the HTNV RNA loads was compared among different organs of the Tg mice, the HTNV RNA loads in liver were lower than those in the spleen (*p* = 0.029) or kidneys (*p* = 0.018) (Figure 6B), indicating that there may be tissue specificity of the peptide FA9-induced CD8^+^ T-cell responses for inhibiting HTNV replication. Together, these data clearly suggest that the inhibition of HTNV replication in the liver, spleen, and kidneys of Tg mice can be mediated by FA9-specific CD8^+^ T-cell responses, which may play an essential role in the partial *in vivo* HTNV clearance in HLA-A2.1/K^b^ Tg mice.

## Discussion

For prevention of infectious diseases, the use of therapeutic or prophylactic vaccines can be a successful method for directing the immune system against specific pathogens. A peptide vaccine with greater efficacy against pathogens would bring about a major improvement in protection of the population against infection (38–40). Our previous findings that the T-cell response was an important component of naturally acquired immunity to control HTNV infection provide the foundation for the development of HTNV peptide vaccines that may combat HTNV by efficiently inducing specific T-cell immunities. In this study, we reported that the HTNV-NP FA9 peptide could be an immunogenic epitope, which was determined based on its binding with HLA-A*0201 molecules and its *in vivo* functional validation. We further provided evidence that immunization with peptide FA9 in HLA-A2.1/K^b^ Tg mice successfully generated reactive CD8^+^ T cell responses that inhibited HTNV infection after HTNV challenge, suggesting that peptide FA9 may be a good candidate for use in the design of HTNV peptide vaccines.

Inactivated vaccines for HTNV in Asia have been proved to boost the humoral responses; however, the titers of HTNV-specific neutralizing antibodies are not sufficiently high in some populations and, therefore, are not expected to be able to effectually limit HTNV amplification and completely prevent infection (13, 14, 41, 42). As such, there is a concerted drive to develop peptide vaccines that can efficaciously induce HTNV-directed T-cell immunity. Previous evidence has illustrated the feasibility, safety, and immunogenicity of the peptide vaccine (43, 44). However, due to MHC restrictions and low immunogenicity, the broad applicability of peptide vaccines for diseases may be currently limited. Several studies on various clinical applications of peptide vaccines have suggested that many potential variables, such as the type and length of peptides, the loading of one or multiple peptides on antigen-presenting cells (APCs) or the route of administration may attribute to the design efficiency of peptide vaccines (25, 39, 40). Selecting the right epitope is the first crucial step in the design of an effective vaccine.

A number of methods have been proposed for the identification of T-cell epitopes based on the binding ability of the predictor peptide to MHC molecules (45, 46). As reported previously, multiple HLA class I-restricted CTL epitopes of HTNV-NP have been identified using overlapping peptides map screen methods in HFRS patients, one of which was the peptide FA9 restricted by HLA-A*02 (21). Our findings also showed that the HTNV-NP FA9 epitope could prime specific CD8^+^ T-cell responses in HLA-A*02 HFRS patients. Intriguingly, patients with higher levels of CD8^+^ T-cell responses to the FA9 epitope maintained mild or moderate HFRS severity (21). Notably, HLA-A*02 allele-restricted epitopes are suggested for further studies because of their potential immunogenicity in Chinese Han populations where HLA-A*02 alleles predominate as high as 29.70% (27, 28). Hence, in this study, we selected the HTNV-NP FA9 peptide for further evaluation of its potential immunogenicity to be used as a candidate for peptide vaccine designs.

We, thus, investigated the structural features of the FA9/HLA-A*0201complex. The electron densities of the FA9 peptide that bound to HLA-A*0201 were unambiguous. The high quality of the overall structure of the pHLA complex allowed for a detailed discussion of the FA9–HLA-A*0201 binding characteristics. The crystal structure analysis offered the opportunity to determine whether the binding sites between FA9 and HLA-A*0201 interact with one another, thereafter evaluating the binding affinity of the peptide FA9 to HLA-A*0201 molecules. In general, the peptides among MHC class I molecules are usually considered to be 8–10 aas long and contain anchor residues for the preferred MHC allele. The exact localization of the anchor residues of the peptide relies on each specific MHC allele. Whether these anchor residues can stably interact with MHC allele-specific pockets is a key determinant in evaluating the affinity of a peptide–MHC (pMHC) molecule (47, 48). In particular, the residue in position 2 from the N termini and the residue on the C termini are the known primary anchor positions for binding of the 9-mer peptides to HLA-A*02 molecules, and the residues at positions 3 and 6 are common secondary anchors in some peptide/HLA-A*02 complexes (49–51). To provide a more accurate analysis of the pHLA, a template GV9 peptide taken directly from the co-crystallized X-ray structure (PDB code 3I6G) was used in our homology structure modeling for the viral pHLA complexes. Our results revealed that the general conformation and position of FA9 within the peptide-binding groove of HLA-A*0201were similar to the structure observed in GV9/HLA-A*0201, and peptide FA9 contains an optimal HLA-A*02-binding motif, that is, Val at the P2 anchor position and Ala at the C-terminal P9 anchor position. However, peptide FA9 bound with HLA-A*0201 molecules via residue Val in position 3 and Leu in position 7 as secondary anchor residues, which appeared to be the eminent feature of peptide FA9. Despite these observed structural differences between the two peptides at the binding sites, the side chain of the P3 residue, occupying identical positions in both the FA9 and GV9 complex, act as the same secondary anchor residues. Thus, peptide FA9 fits well into the binding groove formed at the interface of the pHLA. The stable interaction of peptide FA9 anchor residues with HLA-A*0201 molecules could improve the binding affinity of the complex, which may affect the immunodominance of peptide-specific CTL responses.

It has been reported previously that the biophysical and structural landscape of the pMHC complex plays a critical role in TCR selection (52–54), and the flexibility of the peptide in the MHC groove might affect the responding TCR repertoire (55). Here, we reported that in the middle portion of peptide FA9, the residues Phe1, Pro4, Ile5, Leu6, and Lys8 extruding out of the MHC binding groove may predominantly contribute in interactions with TCR, indicating that the FA9/HLA-A*0201 complex may have ordinary properties as a cell-surface ligand for recognition by T cells. Multiple mechanisms have been proposed for TCR and pMHC recognition and binding (56–58). Because the peptide needs to be presented on the viral-infected cells by MHC molecules at sufficient expression levels to further form stable synapses between the antigen-presenting cells and the effector T cells (59), high TCR to pMHC affinity appears to be a decisive factor for the T-cell immunogenic potential and necessary for specific T cells to completely clear the viruses. In published studies, the kinetic release rate of TCRs from pMHC complexes was found to be most often correlated with T-cell activation (60, 61). The FA9/HLA-A*0201 complex structure determined here implied that the major contacts between TCRs and peptides might be the central hydrophobic residues of the viral peptide. Thus, we speculated that the strong antiviral efficacy of the CTL response induced by FA9 might be primarily derived from the favorable hydrophobic interplay between the central peptide residues and the corresponding hydrophobic residues in the complementary-determining region (CDR) loops of TCRs. However, given that TCR–peptide recognition is mainly determined by the highly flexible and variable CDR loops of TCRs, the TCR–pMHC complex cannot be easily extrapolated (24). Therefore, the definite structure and the affinity for peptide FA9-specific αβTCRs to interact with pHLA complexes have not been fully elucidated, which requires further studies in the future.

It is also noteworthy that functional validation should be performed to ultimately determine immunogenic T-cell epitopes. These structural analyses were then further validated by examining the immunogenicity and antiviral efficacy of peptide FA9 in HLA-A2.1/K^b^ Tg mice. It is important to note that peptides that elicit effective CTL responses must first successfully to be presented by MHC class I molecules. The peptides administered to the mice mimicked the epitopes present on the target cells when associated with the restricting MHC molecule and are, thus, capable of inducing relevant immune responses (25). In the present study, vaccination with the HLA-A*02-restricted HTNV-NP FA9 peptide induced FA9-specific CD8^+^ T-cell responses in HLA-A2.1/K^b^ Tg mice, whereas HLA-B*35-restricted HTNV-NP VY9 peptide vaccination generated relatively poor overall reactivity. In an HTNV challenge trial, there was a significant reduction in both the HTNV antigen level and RNA loads of HTNV in the HLA-A2.1/K^b^ Tg mice vaccinated with the HTNV-NP FA9 peptide compared to unvaccinated Tg mice and compared to the HLA-B*35-restricted VY9 peptide-vaccinated group. These findings indicated that the responses could be inferred as due to the presence of FA9-specific CTLs, which could be proposed to be evidence for natural recognition of the FA9 peptide *in vivo*. Moreover, by examining and comparing the responses to the HLA-A*02-restricted FA9 and HLA-B*35-restricted VY9 peptide in Tg mice, we identified that peptide FA9 could be processed and presented through the classical HLA-A*02-restricted manner, which further primed and boosted specific CTL responses to control HTNV infection *in vivo*. These results are important for the selection of antigenic targets that can be applied in vaccine design to induce protective CD8^+^ T-cell responses. In addition, compared to the external glycoproteins of HTNV, the conservation of the HTNV-NP antigen among different subtypes derived from hantaviruses is generally high (21). Thus, HTNV-NP FA9 epitope that was highly immunogenic in humans might also be a potent immunogen capable of inducing CD8^+^ T-cell responses against HTNV infection in Tg mice, combined with its high conservation, making it an ideal candidate for the development of novel peptide vaccines.

Although antigen-specific IFN-γ production is often used as a biomarker for virus infection, it is possible that additional cytokines might allow more specific or qualitatively different detection of immune responses against virus infection. To further characterize the cellular immune responses specific to HTNV-NP FA9 peptides, additional cytokines and cytotoxic mediators were tested in the splenocytes of immunized Tg mice after *ex vivo* stimulation with the FA9 peptide. In consistent with the data obtained in the ELISPOT assay, immunization with FA9 induced a high percentage of IFN-γ production in the CD8^+^ T cells. TNF-α is a proinflammatory cytokine produced by many types of immunocytes that can enhance Th1 responses, promote CTL activation, and drive antiviral immunity (62). In mice immunized with peptide FA9, an increase in TNF-α expression was observed, as expected. The secretion of granzyme B and the expression of CD107a, which is primarily induced by activated CTLs (63), were also observed to a higher extent in FA9-immunized Tg mice, suggesting that HTNV-NP FA9 peptide-specific CD8^+^ T cells owned a strong cytolytic capacity against HTNV infection. The rapid production of IFN-γ or granules by T cells is important in the defense against HTNV infections. Similar results were observed in Tg mice using HTNV-inactivated vaccine immunization, in which case the percentages of cytokine- or cytotoxic mediator-producing CD8^+^ T cells were significantly higher, whereas peptide VY9-immunized Tg mice had few or no detectable cells expressing cytokines, indicating that the 9-mer peptide presentation requires the matched context of HLA class I molecule.

Notably, the HLA-A2.1/K^b^ Tg mouse model that has been shown to be suitable for the identification of human HLA-A*0201-restricted T-cell epitopes was used in this study (64, 65). In the HTNV challenge experiments, peptide FA9 was as protective as HTNV inactive vaccines, and a similar level of CD8^+^ T-cell responses were observed following both vaccination regimens, suggesting that peptide FA9-primed immune protection is achievable in this mice model. However, there may be some disadvantages of this Tg mouse model for HTNV challenge, as mice are considered a natural reservoir for HTNV but do not develop the disease or symptoms (66). Therefore, we could not evaluate the antivirus effect of the peptide FA9-induced CTL response via a reduction or elimination of disease symptoms. The parameters selected to assess protection were measurements of viral loads in the major organs of the Tg mice. Quantification of HTNV S gene expression encoding the NP with real-time RT-PCR and detection of the HTNV antigen via ELISA in the organs of the mice following HTNV challenge have been shown to be good methods for evaluating HTNV viral loads in mice (67). Based on the peak of virus replication, we chose 3 days post-challenge for detection. Our results indicated that immunization of the HLA-A2.1/K^b^ Tg mice with peptide FA9 followed by HTNV challenge inhibited HTNV replication and enhanced the clearance of HTNV from the liver, spleen, and kidneys of the mice. In the same Tg mice group immunized with peptide FA9, further comparisons showed that the HTNV RNA load in the liver was slightly lower than that in the spleen and kidneys, which was in accordance with the results found in the Tg mice injected with PBS, indicating that even without pre-immunization, the HTNV infection may vary among tissues in Tg mice. In fact, different expressions of tissue-selective homing lymphocyte receptors at the site of antigen exposure and different tissue-specific APCs may lead to different outcomes in the induction of T cells and the impact on sequential antigen-specific responses (68, 69).

In summary, the structural features of the HTNV-NP FA9 peptide/HLA-A*02 molecule complex were analyzed in this study. As far as we know, this is the first report of the crystal structure of the HTNV-NP CTL epitope upon interaction with HLA molecules, through which we confirmed that the HTNV-NP FA9 peptide was a highly immunogenic epitope restricted by HLA-A*02. By challenging HLA-A2.1/K^b^ Tg mice with HTNV *in vivo*, we revealed the *in vivo* immunogenicity of the HTNV-NP peptide FA9 and the antiviral efficiency of FA9-specific CTL responses. The important findings presented here indicated that the HTNV-NP FA9 peptide has high efficacy and immunogenicity for inducing protective immunity and can be used in regimens for further peptide vaccine designs. These observations provide further structural and functional insights regarding T-cell-mediated antiviral immunity in HTNV infection, which may have important implications for both HFRS immunotherapy and HTNV peptide vaccine development. However, the relationship between the TCR-FA9/HLA-A*02 binding and the function of FA9 peptide-specific CTLs is unknown. In follow-up studies, the binding affinity of FA9/HLA-A*02 to specific TCRs should be measured to determine how TCRs interact with their cognate pHLAs to active T cells and induce CTL responses.

## Author Contributions

The study was conceived and experiments designed by BJ, YM, and FZ. The experiments were performed by YM, LFC, and BY. Data were analyzed and interpreted by YM, LFC, BJ, and FZ. YusiZ, CZ, YunZ, KT, and RZ contributed to the reagents, materials, or analysis tools. While YM and BY drafted the paper, LC, KY, BJ, and FZ all critically revised the manuscript for important intellectual content.

## Conflict of Interest Statement

The authors declare that the research was conducted in the absence of any commercial or financial relationships that could be construed as a potential conflict of interest.
