# Supplementary material for: Structure and Function of HLA-A*02-Restricted Hantaan Virus Cytotoxic T-Cell Epitope That Mediates Effective Protective Responses in HLA-A2.1/Kb Transgenic Mice
Source: Front Immunol. 2016 Aug 8;7:298. doi: 10.3389/fimmu.2016.00298 (PMC4976285; doi:10.3389/fimmu.2016.00298)
Supplement: Supplementary file 1 [file data_sheet_1.zip › Supplementary Data/Supplementary Material--Figure Legends.docx]

**Supplementary Material-Figure Legends**

**Figure S1 The purification of MHC recombinant protein.** 12% SDS-PAGE electrophoresis and coomassie blue staining were used to detect the molecular weight of the HLA-A*0201 heavy chain and β2 microglobulin (β2 m).

**Figure S2 Crystal image of the HTNV-NP FA9 peptide/HLA-A*0201 complex.** **(A)** Early screen crystal image of the FA9/HLA-A*0201 complex acquired using the crystallization kit with the PEGRx 1 HR2-082 35# condition. **(B)** An image of the initial 2 months of growth crystal of the FA9 /HLA-A*0201 complex.

**Figure S3 Preliminary detection of HTNV viremia in the major organs of HLA-A2.1/K^b^ Tg mice.** HTNV antigen detection via ELISA in six organs, including the liver, spleen, kidneys, lungs, cerebrum and heart, in naïve Tg mice or Tg mice challenged with HTNV.
