# Supplementary figures and images for: Structure and Function of HLA-A*02-Restricted Hantaan Virus Cytotoxic T-Cell Epitope That Mediates Effective Protective Responses in HLA-A2.1/Kb Transgenic Mice
Source: Front Immunol. 2016 Aug 8;7:298. doi: 10.3389/fimmu.2016.00298 (PMC4976285; doi:10.3389/fimmu.2016.00298)

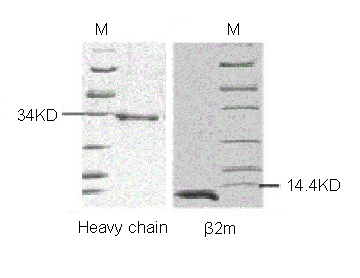

Supplement: Supplementary file 1 [file data_sheet_1.zip › Supplementary Data/Figure S1.tif]

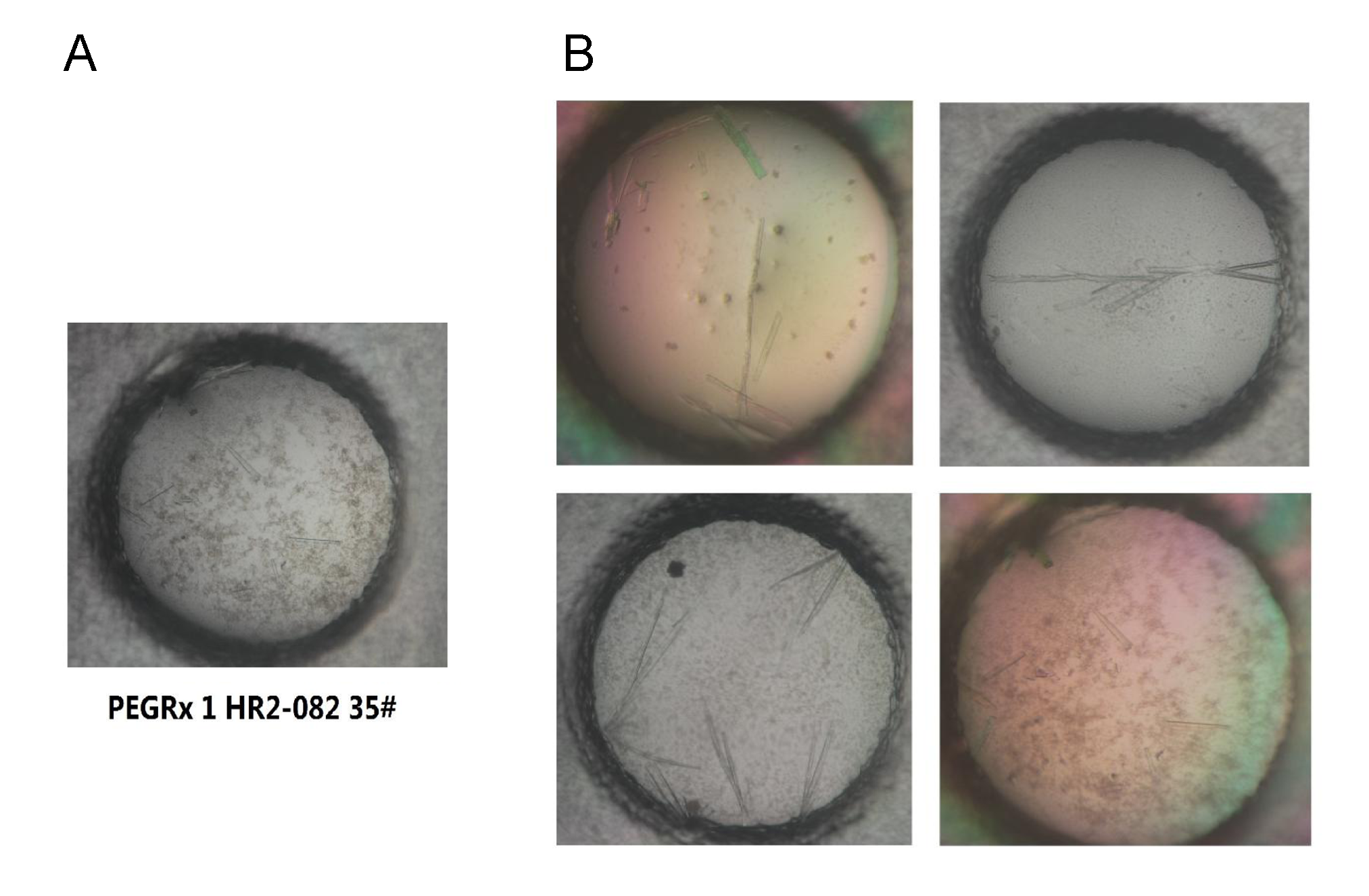

Supplement: Supplementary file 1 [file data_sheet_1.zip › Supplementary Data/Figure S2.tif]

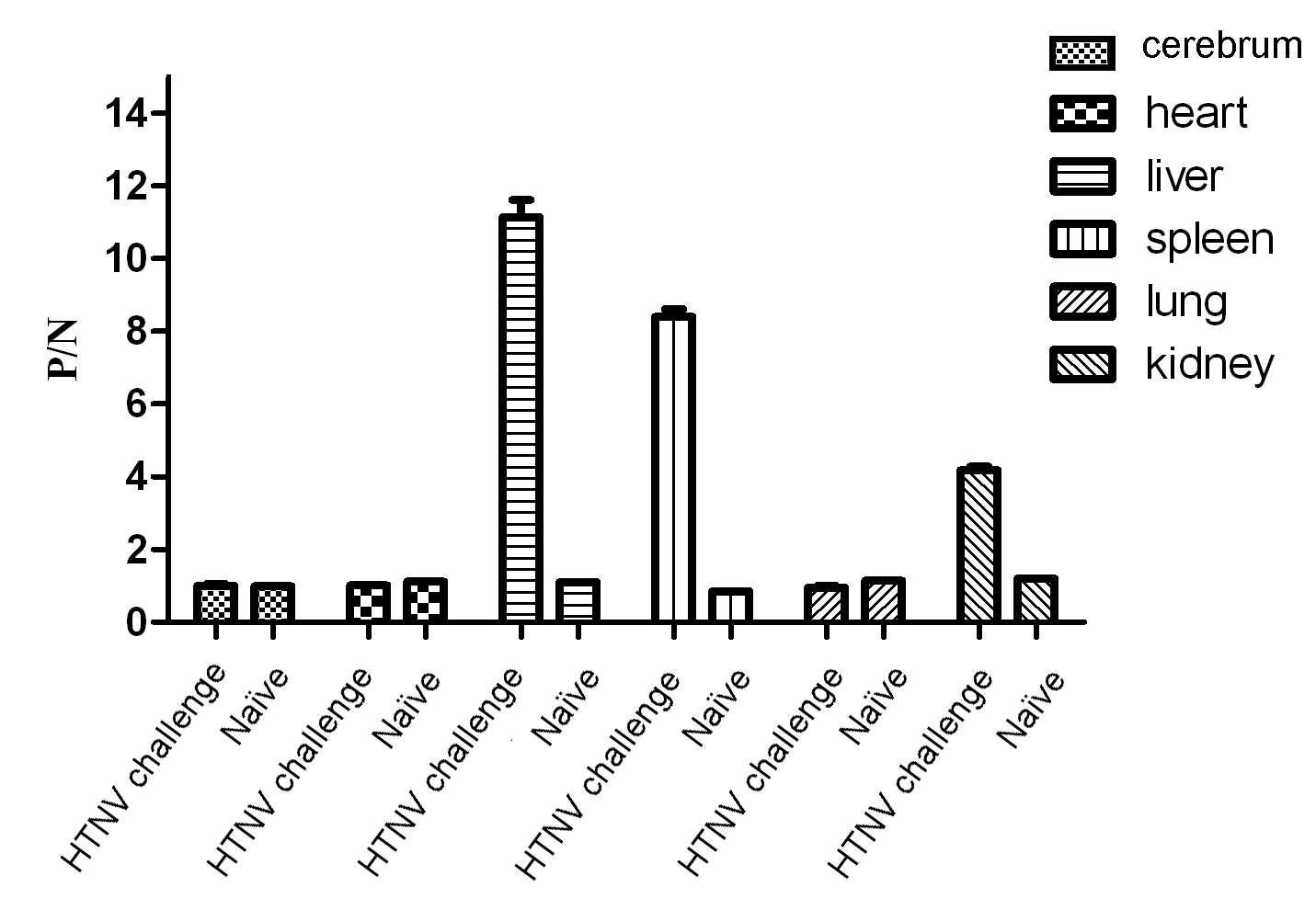

Supplement: Supplementary file 1 [file data_sheet_1.zip › Supplementary Data/Figure S3.tif]
